# Phytantriol-Based Berberine-Loaded Liquid Crystalline Nanoparticles Attenuate Inflammation and Oxidative Stress in Lipopolysaccharide-Induced RAW264.7 Macrophages

**DOI:** 10.3390/nano12234312

**Published:** 2022-12-05

**Authors:** Abdullah M. Alnuqaydan, Abdulmajeed G. Almutary, Mohd Azam, Bikash Manandhar, Gabriele De Rubis, Thiagarajan Madheswaran, Keshav Raj Paudel, Philip M. Hansbro, Dinesh Kumar Chellappan, Kamal Dua

**Affiliations:** 1 Department of Medical Biotechnology, College of Applied Medical Sciences, Qassim University, Buraidah 51452, Saudi Arabia; 2 Department of Medical Laboratories, College of Applied Medical Sciences, Qassim University, Buraidah 51452, Saudi Arabia; 3 Discipline of Pharmacy, Graduate School of Health, University of Technology Sydney, Sydney, NSW 2007, Australia; 4 Faculty of Health, Australian Research Centre in Complementary & Integrative Medicine, University of Technology Sydney, Ultimo, NSW 2007, Australia; 5 Department of Pharmaceutical Technology, School of Pharmacy, International Medical University, Kuala Lumpur 57000, Malaysia; 6 Centre for Inflammation, Faculty of Science, School of Life Sciences, Centenary Institute and University of Technology Sydney, Sydney, NSW 2007, Australia; 7 Department of Life Sciences, School of Pharmacy, International Medical University, Kuala Lumpur 57000, Malaysia

**Keywords:** berberine, liquid crystalline nanoparticles, inflammation, oxidative stress, macrophages, LPS, advanced drug delivery, chronic inflammatory diseases

## Abstract

Inflammation and oxidative stress are interrelated processes that represent the underlying causes of several chronic inflammatory diseases that include asthma, cystic fibrosis, chronic obstructive pulmonary disease (COPD), allergies, diabetes, and cardiovascular diseases. Macrophages are key initiators of inflammatory processes in the body. When triggered by a stimulus such as bacterial lipopolysaccharides (LPS), these cells secrete inflammatory cytokines namely TNF-α that orchestrate the cellular inflammatory process. Simultaneously, pro-inflammatory stimuli induce the upregulation of inducible nitric oxide synthase (iNOS) which catalyzes the generation of high levels of nitric oxide (NO). This, together with high concentrations of reactive oxygen species (ROS) produced by macrophages, mediate oxidative stress which, in turn, exacerbates inflammation in a feedback loop, resulting in the pathogenesis of several chronic inflammatory diseases. Berberine is a phytochemical embedded with potent in vitro anti-inflammatory and antioxidant properties, whose therapeutic application is hindered by poor solubility and bioavailability. For this reason, large doses of berberine need to be administered to achieve the desired pharmacological effect, which may result in toxicity. Encapsulation of such a drug in liquid crystalline nanoparticles (LCNs) represents a viable strategy to overcome these limitations. We encapsulated berberine in phytantriol-based LCNs (BP-LCNs) and tested the antioxidant and anti-inflammatory activities of BP-LCNs in vitro on LPS-induced mouse RAW264.7 macrophages. BP-LCNs showed potent anti-inflammatory and antioxidant activities, with significant reduction in the gene expressions of TNF-α and iNOS, followed by concomitant reduction of ROS and NO production at a concentration of 2.5 µM, which is lower than the concentration of free berberine concentration required to achieve similar effects as reported elsewhere. Furthermore, we provide evidence for the suitability for BP-LCNs both as an antioxidant and as an anti-inflammatory agent with potential application in the therapy of chronic inflammatory diseases.

## 1. Introduction

Inflammation is a complex physiological reaction occurring in various tissues in response to a plethora of endogenous and exogenous stimuli, that may include pathogens such as viruses and bacteria, as well as exposure to toxic chemicals, allergens, and radiations [1]. Physiologically, the primary aim of inflammation is to eliminate the initial damaging stimulus. However, prolonged and dysregulated inflammation eventually leads to chronic inflammatory diseases such as diabetes, cardiovascular diseases, arthritis, joint diseases, allergies, and chronic respiratory diseases. Among these asthma, chronic obstructive pulmonary disease (COPD), and lung cancer are of utmost significance [2,3,4,5]. Therefore, chronic inflammation constitutes to be the main etiological factor for a wide range of diseases. Inflammation is mediated by different classes of inflammatory cells. Among these, macrophages and neutrophils take part in the initiation of the inflammatory cascade, and hence play significant roles in the pathogenesis of the aforementioned pathophysiological features through the secretion of various inflammatory mediators that may include the inflammatory cytokines tumor necrosis factor-α (TNF-α), Interleukin 6 (IL-6), and IL-1β [6,7]. It is well known that lipopolysaccharides (LPS), a bacterial toxin generally found in the cell membrane of Gram-negative bacteria cause exacerbated inflammatory cascade primarily in cells including macrophages [8,9].

Oxidative stress is a pathogenic mechanism that results when the balance between the generation of oxidative agents, namely reactive oxygen species (ROS), reactive nitrogen intermediates (RNI), and their elimination through biological antioxidants, other defense mechanisms has been compromised [10,11,12]. ROS and RNI are physiologically produced by cells and, at low concentrations, function as important mediators and signaling molecules involved in the maintenance of cell homeostasis, and oxidative stress. Accumulation of these species leads to the damage of cellular components such as DNA, lipids, and proteins [1]. For this reason, oxidative stress is a potent inducer of inflammation, and it is known to exacerbate inflammatory processes, leading to the aggravation of chronic inflammatory diseases [1,10,13]. Moreover, proinflammatory triggers such as LPS are known to induce the generation of high levels of ROS and nitric oxide (NO) in macrophages and in other inflammatory cells [14,15]. NO is one of the principal RNI which is generated by macrophages, and other inflammatory cells, upon activation of the inducible NO synthase (iNOS). iNOS is a key enzyme that is mediated by macrophages in the inflammatory cascade [16]. ROS are highly unstable chemical species that are known to attack NO and convert it into peroxynitrite, a toxic intermediate that causes apoptosis in many cell types, further exacerbating the severity of inflammation [14].

Considering the extent of the cross-talk between inflammation and oxidative stress, these two processes are tightly interrelated and, together, play pivotal roles in the pathogenesis of chronic diseases [17]. The strictly interdependent relationship between oxidative stress and inflammation has been recently proposed as a possible explanation for the “antioxidant paradox”, which is referred to the observation that dietary administration of large doses of antioxidants is often ineffective in the prevention or treatment of diseases where the disease pathogenesis is heavily influenced by oxidative stress [17,18]. To address this concern, the use of compounds endowed with both antioxidant and anti-inflammatory properties could be beneficial.

A valuable source for potential therapeutic agents, in this context, is represented by natural sources such as traditional medicinal plants, which have provided several promising novel phytochemical compounds such as rutin [19], boswellic acids [20], curcumin [21], and nobiletin to name a few [22]. Berberine, an isoquinoline alkaloid molecule extracted from the root, stem bark, and rhizomes of plants belonging to the genus Berberis, is widely known for its antioxidant, anti-inflammatory, and anticancer properties [23,24,25,26,27,28,29,30]. It has also been shown to improve the lipid and glucose profile when administered in association with silymarin, thus promoting cardiometabolic health [31].

Despite the encouraging beneficial activities of berberine against many illnesses, its therapeutic application, similar to that of many other promising phytochemicals, is severely limited by issues such as poor oral bioavailability and limited intestinal absorption, which hamper in vivo efficacy and clinical translation [32,33,34]. A valuable approach to overcome these limitations is to employ advanced drug delivery methods, such as encapsulation of therapeutic molecules in advanced nanoparticle (NP)-based systems [35]. Among the many currently available advanced nanoformulations, liquid crystalline nanoparticle (LCNs) based delivery represents a highly versatile tool with the substantial capability of enhancing the bioavailability and stability of therapeutic moieties [36]. In this context, our research group has recently demonstrated significant in vitro anticancer action of berberine-loaded LCN formulation against non-small-cell lung cancer (NSCLC) cells [37,38]. Furthermore, we showed that a berberine-phytantriol LCN formulation possessed strong in vitro cytotoxic and anti-migratory activity against NSCLC [39]. In another study, we showed that berberine-loaded LCNs were highly effective in attenuating oxidative stress, senescence, and inflammation triggered by cigarette smoke in an in vitro model of COPD obtained by exposing mouse macrophage cells (RAW264.7) and human broncho-epithelial cells (16HBE) to cigarette smoke extract [40].

In the current study, we explored the protective activity of the berberine-phytantriol LCN formulation (BP-LCN) against LPS-induced inflammation and oxidative stress on mouse RAW264.7 macrophages. We demonstrate that BP-LCNs significantly reduce LPS-induced ROS production, along with the upregulation of the pro-inflammatory cytokines TNF-α, IL-6, and IL-1β. Furthermore, BP-LCNs were found to reduce LPS-induced iNOS expression, with a concomitant reduction of NO production. The results of our study confirm the dual antioxidant/anti-inflammatory activity of berberine, providing valuable proof of the applicability of berberine-loaded LCNs as a therapeutic agent in chronic inflammatory conditions.

## 2. Materials and Methods

### 2.1. Cell Culture and Formulation Aspects of BP-LCNs

A Dulbecco’s modified Eagle’s medium (DMEM) (Lonza, Basel, Switzerland) was employed to culture the procured RAW 264.7 mouse macrophages (ATCC, Manassas, VA, USA). The entire process was performed in a humidified incubator. The incubator was monitored at a temperature of 37 °C with 5% CO_2_. Furthermore, the medium also contained 5% (*v*/*v*) foetal bovine serum (FBS) (Lonza, Basel, Switzerland) along with 1% (*v*/*v*) penicillin-streptomycin (Lonza, Basel, Switzerland). The experiments were carried out in the DMEM both in the presence and absence of berberine-phytantriol LCNs maintained at a temperature of 37 °C with 5% CO2. BP-LCN formulations were prepared as described previously [39].

### 2.2. Cell Viability Determination

The determination of cell viability was conducted in RAW 264.7 cells using MTT reagent (3-(4,5-Dimethylthiazol-2-yl)-2,5-diphenyltetrazolium bromide, Sigma-Aldrich, St. Louis, MO, USA) as described previously [41]. Briefly, seeding of the RAW 264.7 cells was carried out at a density of 20,000 cells/well in a clear-bottomed, transparent 96-welled plate. This mixture was incubated at 37 °C for a duration of 24 h. Subsequently, these cells were incubated for 1 h in the presence of lipopolysaccharide (LPS) obtained from Escherichia coli (with a final concentration of 1 µg/mL, Merck, Kenilworth, NJ, USA). An additional 24 h incubation was allowed for the cells both with and without the BP-LCNs. Finally, the working concentrations employed were 0.5, 1.0, 2.5, 5.0, or 10.0 µM. Subsequently, to the wells, 250 µg/mL MTT reagent was incorporated. The mixture was then incubated further for 4 h, after which the supernatant was removed with caution. Dimethyl sulfoxide (DMSO) (Merck, Kenilworth, NJ, USA) was employed to solubilize the developed formazan crystals. This was followed by measuring the absorbance levels of the mixture. The wavelength employed was 540 nm. The entire operation was performed with a POLARstar Omega microplate reader (BMG Labtech, Ortenberg, Germany).

### 2.3. Determination of ROS Generation

Generation of cellular ROS was determined with 2′,7′-dihydrodichlorofluorescin diacetate (DCF-DA, Merck, Kenilworth, NJ, USA) using a fluorescence plate reader and fluorescence microscopy, as described previously [40].

#### 2.3.1. ROS Determination by Fluorescence Plate Reader

A black 96-welled plate (Greiner Bio-One GmbH, Frickenhausen, Germany) was employed to seed the RAW264.7 cells. The cells were seeded for a 24 h duration. Subsequently, these cells were subjected to preincubation for 1 h with LPS (final concentration 1 µg/mL). The mixture was then incubated with or without BP-LCNs for a period of 24 h with an eventual concentration of 1.0 or 2.5 µM respectively. This was followed by a 30-min incubation at room temperature under a dark condition in the presence of DCF-DA (final concentration 10 µM). The fluorescence intensity was determined at an excitation wavelength of 488 nm. The emission wavelength employed was 525 nm. The entire operation was performed with a FLUOstar Omega (BMG LABTECH Pty Ltd., Victoria, Australia).

#### 2.3.2. ROS Determination by Fluorescence Microscopy

RAW264.7 cells were grown on a coverslip of a 6-welled plate (Griener Bio-One GmbH). Post overnight incubation, the cells were preincubated with 1 µg/mL LPS for 1 h. This was followed by the incubation for 24 h with or without BP-LCNs (1.0 or 2.5 µM respectively). This was followed by washing of the cells with phosphate-buffered saline (PBS, 2X washes). The mixture was subsequently subjected to incubation with 10 μM of DCF-DA in dark at room temperature for a duration of 30 min. Then PBS was used to wash the cells (2X) and the fluorescence images were captured at 20× magnification using a fluorescence microscope (Zeiss Axio Imager Z2, Oberkochen, Germany).

### 2.4. Determination of NO Production with Griess Reagent

The levels of NO secretion by RAW264.7 was determined using a modified Griess reagent (Merck). A transparent, clear-bottom 96-well plate was employed for seeding the RAW264.7 cells. A total of 24 h after incubation, the cells were subjected to pre-incubation for 1 h with 1 µg/mL LPS, which was then incubated for 24 h with or without the BP-LCNs (1.0 or 2.5 µM respectively). The culture supernatants were then collected, and 150 µL of the supernatant was added onto a clear bottom, transparent 96-well plate and treated with Griess reagent (1:1 (*v*/*v*) ratio). The samples were then subjected to incubation for 30 min duration at room temperature in dark conditions. The relative levels of NO in the form of nitrite were recorded by measuring the absorbance at 540 nm (excitation wavelength) using a TECAN Infinite M1000 plate reader (Tecan Trading AG, Männedorf, Switzerland). The absorbance of the blank (unconditioned cell culture media) was subtracted from the absorbance of each sample and variations of NO levels were reported as percentages compared to the control (untreated) group.

### 2.5. Real-Time Quantitative Polymerase Chain Reaction (PCR)

The experiment was performed with 6-well plates which were used to grow the RAW264.7 cells. After reaching 80% confluency, preincubation was carried out with LPS (1 µg/mL) for a duration of 1 h. The mixture was then incubated for 24 h both with and without BP-LCNs (2.5 µM). Subsequently, PBS was used to wash the cells (2X), and were lysed using the TRI reagent (Merck). The next step involved the total isolation of RNA [39]. The purity and the concentration of RNA were measured by Nanodrop One (Thermo Fisher Scientific, North Ryde, NSW, Australia).

A quantitative reverse transcription PCR technique was employed to measure the mRNA levels [38]. Briefly, the mixture was subjected to DNase treatment with a DNase I kit (Merck), 1 µg of RNA was used to synthesize cDNA by reverse transcription using a Mastercycler Nexus GSX1 thermal cycler (Eppendorf, Hamburg, Germany) via subsequent steps of denaturation (65 °C, 15 min) and annealing (25 °C, 10 min). After this, reverse transcription at 37 °C for 50 min was performed followed by the inactivation of enzymes at 70 °C, for 15 min were performed. The reaction mix contained random primers (500 ng/µL), dNTPs (10 mM), MMLV reaction buffer (1×), and DTT (100 mM). Real-time qPCR was conducted on 25 ng cDNA with a CFX96 real-time PCR detection system (Bio-Rad, Hercules, CA, USA) using iTaq Universal SYBR Green supermix (1×, Bio-Rad, Hercules, CA, USA) and 0.5 µM of each of the forward and reverse primers for TNF-α (Forward: TCTGTCTACTGAACTTCGGGGTGA; Reverse: TTGTCTTTGAGATCCATGCCGTT), IL-1β (Forward: TGGGATCCTCTCCAGCCAAGC; Reverse: AGCCCTTCATCTTTTGGGGTCCG), IL-6 (Forward: AGAAAACAATCTGAAACTTCCAGAGAT; Reverse: GAAGACCAGAGGAAATTTTCAATAGG), iNOS (Forward: AGCGAGGAGCAGGTGGAAGACT; Reverse: CCATAGGAAAAGACTGCACCGAA), and HPRT (Forward: AGGCCAGACTTTGTTGGATTTGAA; Reverse: CAACTTGCGCTCATCTTAGGCTTT).

### 2.6. Statistical Analysis

All data were presented as Mean ± SEM. To check for normal distribution of data, the data points for each group were plotted on a histogram and visually inspected. Upon visual confirmation of normal distribution, data were analyzed by 1-way ANOVA, followed by Dunnett multiple comparisons test, using GraphPad Prism (v.9.4, GraphPad Software, San Diego, CA, USA). The pairwise comparisons of a 2-tailed *p*-value < 0.05 were considered statistically significant.

## 3. Results

### 3.1. Determination of Optimum Dose of BP-LCNs for Treatment in LPS-Induced RAW 264.7 Cells

**Figure 1:**
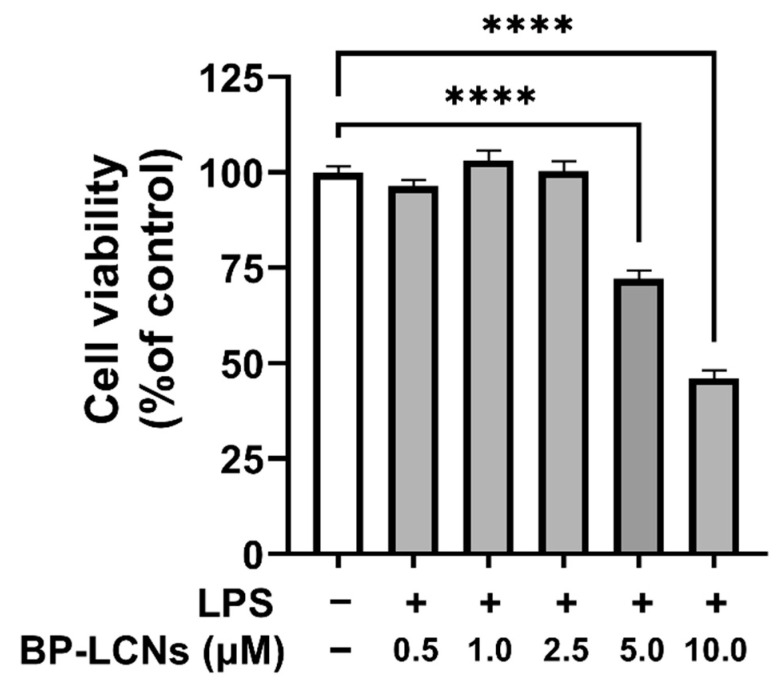
Effect of BP-LCNs on the cell viability of LPS-induced RAW 264.7 cells. RAW 264.7 cells were pre-incubated for 1 h in the presence of LPS (final concentration 1 µg/mL), then incubated for 24 h in the absence or presence of BP-LCNs (final concentration 0.5, 1.0, 2.5, 5.0 or 10.0 µM). MTT reagent was added to each well, the cells were incubated for a further 4 h and the formazan crystals were dissolved in DMSO. The cell viability was determined by measuring absorbance at 540 nm with a microplate reader and the cell viability was normalized as a percentage of control cells. The results are Mean ± SEM of three independent experiments (**** *p* < 0.0001).

The MTT assay results showed that BP-LCNs did not induce cell toxicity on LPS-induced RAW 264.7 cells up to a concentration of 2.5 µM (Figure 1). However, the cell viability of RAW 264.7 cells decreased significantly when they were incubated in the presence of BP-LCNs at 5 and 10 µM (Figure 1, *p* < 0.0001 for both against the control). Hence, the non-toxic doses of 1.0 and/or 2.5 µM BP-LCNs were used in the subsequent experiments.

### 3.2. BP-LCNs Inhibit ROS Generation in a Dose-Dependent Manner

**Figure 2:**
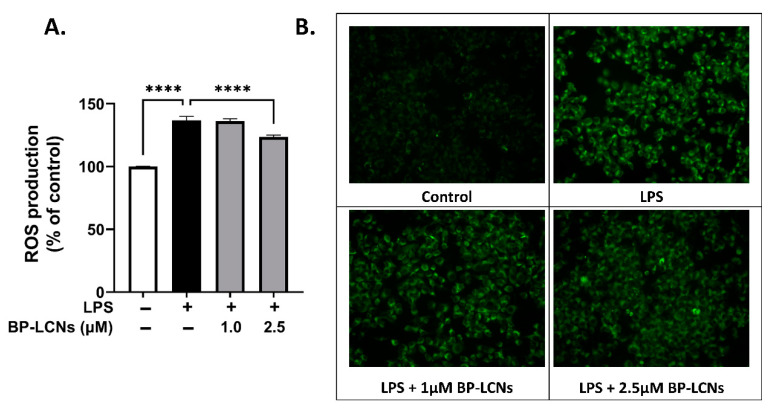
Effect of BP-LCNs on LPS-induced ROS production in RAW 264.7 cells. RAW 264.7 cells were pre-incubated for 1 h in the presence of LPS (final concentration 1 µg/mL), then incubated for 24 h in the absence or presence of BP-LCNs (final concentration 1.0 or 2.5 µM). The ROS generation was determined semi-quantitatively and qualitatively by measuring the fluorescence intensity of DCF-DA using a fluorescence plate reader (**A**) and fluorescence microscopy (**B**). The values in (**A**) represent the Mean ± SEM of three independent experiments (**** *p* < 0.0001).

The anti-oxidative potential of BP-LCNs was assessed by measuring ROS levels in LPS-induced RAW 264.7 cells. The LPS induction increased oxidative stress by 38% increase in ROS production (Figure 2A, *p* < 0.0001 against control). The ROS levels were not affected by BP-LCNs at a concentration of 1 µM (Figure 2A). However, the inclusion of BP-LCNs at a concentration of 2.5 µM decreased ROS production by 10% (Figure 2A, *p* < 0.0001 against cells treated with LPS alone). The increase in ROS production by LPS induction was apparent due to the increased fluorescence of DCF-DA in LPS-induced RAW 264.7 cells (Figure 2B). As observed in the fluorescence images, the inclusion of BP-LCNs in the incubation inhibited LPS-induced NO production at both 1.0 and 2.5 µM concentrations (Figure 2B).

### 3.3. BP-LCNs Inhibit NO Generation

**Figure 3:**
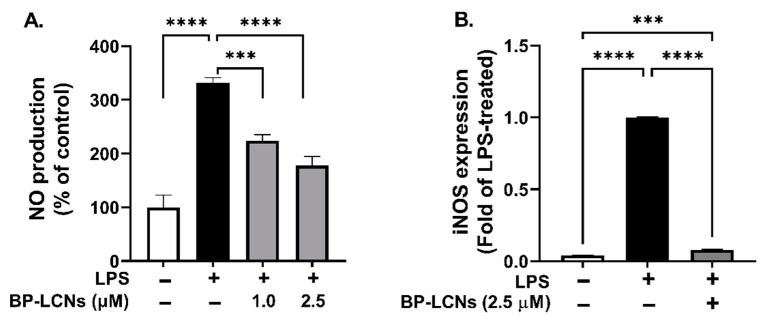
Effect of BP-LCNs on LPS-induced NO production and iNOS mRNA expression in RAW 264.7 cells. RAW 264.7 cells were pre-incubated for 1 h in the presence of LPS (final concentration 1 µg/mL), then incubated for 24 h in the absence or presence of BP-LCNs (final concentration 1.0 or 2.5 µM). The NO production in the culture supernatant was determined by using Griess reagent and measuring the absorbance with a fluorescence plate reader (**A**). The mRNA levels of iNOS (**B**) were determined by RT-qPCR as described in Materials and Methods Section 2.5. The values in (**A**,**B**) are Mean ± SEM of three independent experiments (*** *p* < 0.001; **** *p* < 0.0001).

The potential of BP-LCNs to inhibit NO production was assessed by measuring NO levels in the culture supernatant and mRNA levels of iNOS in LPS-induced RAW 264.7 cells. LPS induction increased NO production 3.3-fold (Figure 3A, *p* < 0.0001 against control). The inclusion of BP-LCNs at concentrations of 1 µM and 2.5 µM significantly decreased NO production by 32% (Figure 3A, *p* < 0.001 against control) and 46% (Figure 3A, *p* < 0.0001 against control), respectively.

The underlying mechanism may be explained by BP-LCNs-mediated regulation of iNOS gene expression in LPS-induced RAW 264.7 cells. The mRNA levels of LPS-treated RAW 264.7 cells increased by 23-fold compared to the control (Figure 3B, *p* < 0.0001). However, the inclusion of BP-LCNs in the incubation decreased the iNOS mRNA levels by 0.92-fold compared to LPS alone-treated RAW 264.7 cells (Figure 3B, *p* < 0.0001). The iNOS mRNA levels in BP-LCNs-treated RAW 264.7 cells were comparable to the values of the control cells (Figure 3B).

### 3.4. BP-LCNs Inhibit mRNA Levels of the Pro-Inflammatory Cytokines TNF-α, IL-6, and IL-1β

**Figure 4:**
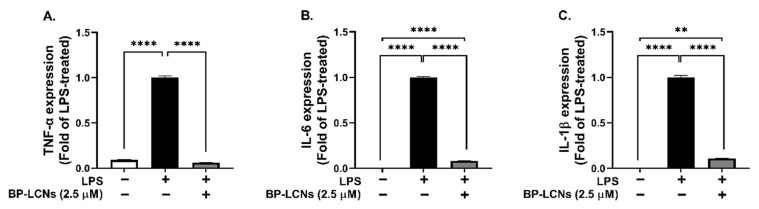
Effect of BP-LCNs on mRNA expression of LPS-induced pro-inflammatory cytokines TNF-α (**A**), IL-6 (**B**), and IL-1β (**C**) in RAW 264.7 cells. RAW 264.7 cells were pre-incubated for 1 h in the presence of LPS (final concentration 1 µg/mL), then incubated for 24 h in the absence or presence of BP-LCNs (final concentration 2.5 µM). Total RNA was extracted using TRI reagent, cDNA synthesized by reverse transcription, and mRNA levels for TNF-α, IL-6, and IL-1β were determined by quantitative PCR with SYBR green. The values shown are Mean ± SEM of three independent experiments (** *p* < 0.01; **** *p* < 0.0001).

The anti-inflammatory potential of BP-LCNs was assessed on LPS-induced RAW 264.7 cells by measuring the mRNA levels of pro-inflammatory cytokines TNF-α, IL-6, and IL-1β. The LPS induction in RAW 264.7 cells significantly increased the mRNA levels of these cytokines compared to the control (Figure 4, *p* < 0.0001). The inclusion of BP-LCNs in the incubation significantly decreased the mRNA levels of these cytokines compared to the control (Figure 4A–C, *p* < 0.0001 against LPS-only treated cells). Thus, the LPS-induced increase in the mRNA level of these cytokines was significantly inhibited in the presence of BP-LCNs, suggesting a strong anti-inflammatory potential of BP-LCNs. The LPS-induced mRNA levels of TNF-α were decreased by BP-LCNs to levels comparable to the control cells (Figure 4A).

## 4. Discussion

Inflammation and oxidative stress are two intimately interrelated processes that represent fundamental aetiological factors for many chronic inflammatory diseases. One of the many pathways that initiate inflammatory responses involves the stimulation of macrophages with the bacterial molecule LPS, which initiates a signaling cascade mediated by the toll-like receptor 4 (TLR4). This activates downstream signaling pathways resulting, among many outcomes, in increased secretion of pro-inflammatory cytokines, increased ROS production, as well as increased iNOS expression that, in turn, generates higher levels of NO [14].

Here, we demonstrated significant anti-inflammatory and antioxidant activity of BP-LCNs in LPS-induced RAW264.7 mouse macrophages, which was mediated by inhibition of the transcription of the pro-inflammatory cytokines TNF-α, IL-6, and IL-1β, as well as through reduction of ROS production, inhibition of iNOS expression and subsequent reduction of LPS-induced NO production.

Despite the many demonstrated clinical benefits, berberine is characterized by very low water solubility [42]. This, together with the fact that it is poorly absorbed by the gastrointestinal tract, results in its characteristic poor bioavailability (0.68% in rats) [43], which severely hampers its therapeutic use. Poor solubility and relatively low bioavailability are common characteristics across a wide range of medicinally active phytochemicals [44]. Due to these limitations, high doses of berberine should be administered to achieve the relevant pharmacological activity, but this might not be feasible due to the possibility of toxic effects, which are more evident when using pure berberine compared to plant extracts or plant fractions containing it [45]. For this reason, the use of nanotechnology-based innovative drug delivery technologies involving the encapsulation of berberine in LCNs is a viable strategy to improve its physicochemical properties, enhancing its therapeutic efficacy and potency.

In this study, BP-LCNs demonstrated anti-inflammatory and antioxidant activity when used at an equivalent berberine concentration of 2.5 µM. The low effective berberine concentration needed to exert this effect is concordant with our previous study, in which we demonstrated that berberine encapsulated in LCNs has significant dose-dependent antioxidant activity at concentrations ranging between 1 and 5 µM, and anti-inflammatory and anti-senescence activity at 5 µM, on both RAW264.7 and 16HBE human broncho-epithelial cells exposed to cigarette smoke extract [40]. In comparison, a similar anti-inflammatory effect was reported when using concentrations of free berberine powder between 10 and 100 µM [25,30,46]. This represents a clear demonstration of the advantages of formulating poorly soluble molecular moieties in advanced drug delivery systems such as LCNs to overcome common limitations affecting many phytochemicals. Taken together with our previous results [40], the findings reported in the present study demonstrate how berberine-encapsulated LCNs are capable of counteracting inflammation and oxidative stress induced by different stimuli, namely cigarette smoke, and LPS. This inspires further research to provide a mechanistic explanation of the molecular pathways impacted by berberine that lead to its therapeutic activity. In particular, it would be interesting to investigate whether these activities are exerted by impacting signaling pathways such as NF-kB, MAPKinase, and the NLRP3 inflammasome pathway. These investigations will represent the core of our future studies on the characterization of the therapeutic activity of BP-LCNs.

Although we observed promising antioxidant and anti-inflammatory activity of BP-LCNs, a limitation of our study lies in the fact that we only investigated the expression of TNF-α, IL-6, and IL-1β mRNA to assess the anti-inflammatory activity of BP-LCNs. Although the inhibitory effect of berberine on these fundamental cytokines is in agreement with studies from our laboratory and other research groups [40,47,48], it would be useful to extend these findings to better understand the effect of our BP-LCNs on other cytokines and pro- and anti-inflammatory mediators. Another limitation lies in the fact that the experiments were entirely performed in vitro. To complement our findings, it would be interesting to investigate the activity of BP-LCNs in preclinical animal models of diseases for which inflammation and oxidative stress represent an important aetiological factor such as cancer, cardiovascular diseases, arthritis, chronic respiratory diseases, and others. Considering the versatility of LCNs as drug delivery systems for inflammatory lung diseases, which allow direct lung delivery of therapeutic moieties via inhalation [36], it would be particularly interesting to test the in vivo activity of our BP-LCNs on animal models of chronic respiratory diseases such as asthma [49,50] and COPD [51].

Despite this limitation, this study suggests that encapsulation of berberine in LCNs has potent dual antioxidant and anti-inflammatory activity at relatively low berberine concentrations, resulting in improved physicochemical characteristics and potentially reduced toxic effects.

## 5. Conclusions

Our study demonstrates the advantages of the LCN-based formulation of berberine, which exerted potent in vitro anti-inflammatory and antioxidant activity in LPS-induced RAW246.7 macrophages at lower concentrations compared to free berberine. These effects were mediated by inhibition of TNF-α, IL-6, IL-1β, and iNOS gene expression and simultaneous reduction of ROS and NO production. In conclusion, these results provide proof of the validity of BP-LCNs as a new candidate for further development as a therapeutic strategy for chronic inflammatory diseases. However, to allow translation into clinical practice, these findings need to be validated by preclinical and clinical studies.

## Data Availability

Data may be made available on request from the corresponding author.

## Abbreviations

BP-LCNs: Berberine-Phytantriol Liquid Crystalline Nanoparticles; COPD: Chronic Obstructive Pulmonary Disease; DCF-DA: 2′,7′-dihydrodichlorofluorescin diacetate; DMEM: Dulbecco’s modified Eagle’s medium; DMSO: dimethyl sulfoxide; IL: Interleukin; iNOS: Inducible Nitric Oxide Synthase; LCN: Liquid Crystalline Nanoparticles; LPS: Lipopolysaccharide; NO: Nitric Oxide; NSCLC: non-small-cell lung cancer; RNI: reactive nitrogen intermediates; ROS: Reactive Oxygen Species; TNF-α: Tumour Necrosis Factor-α; TLR4: Toll-Like Receptor 4.
